# S137 Phosphorylation of Profilin 1 Is an Important Signaling Event in Breast Cancer Progression

**DOI:** 10.1371/journal.pone.0103868

**Published:** 2014-08-01

**Authors:** Wasia Rizwani, Aneesa Fasim, Deepshikha Sharma, Divya J. Reddy, Nabil A. M. Bin Omar, Surya S. Singh

**Affiliations:** Department of Biochemistry, Osmania University, Hyderabad, A.P., India; West Virginia University, United States of America

## Abstract

**Background:**

Profilins are actin-modulating proteins regulating many intracellular functions based on their multiple and diverse ligand interactions. They have been implicated to play a role in many pathological conditions such as allergies, cardiovascular diseases, muscular atrophy, diabetes, dementia and cancer. Post-translational modifications of profilin 1 can alter its properties and subsequently its function in a cell. In the present study, we identify the importance of phosphorylation of profilin 1 at serine 137 (S137) residue in breast cancer progression.

**Methods/Principal Findings:**

We found elevated profilin 1 (PFN) in human breast cancer tissues when compared to adjacent normal tissues. Overexpression of wild-type profilin 1 (PFN-WT) in breast cancer MCF7 cells made them more migratory, invasive and adherent independent in comparison to empty vector transfected cells. Mutation in serine phosphorylation site (S137) of profilin 1 (PFN-S137A) significantly abrogated these properties. Mutation affecting actin-binding ability (PFN-R74E) of profilin 1 enhanced its tumorigenic function whereas mutation affecting its poly-L-proline binding function (PFN-H133S) alleviated these mechanisms in breast cancer cells. PFN-WT was found to activate matrix metalloproteinases by zymography, MMP2 and MMP9 in presence of PDBu (phorbol 12, 13 dibutyrate, PI3K agonist) to enhance migration and invasion in MCF7 cells while PFN-S137A did not. Phosphorylation increased migration and invasion in other mutants of profilin 1. Nuclear profilin levels also increased in the presence of PDBu.

**Conclusions:**

Previous studies show that profilin could be executing a dual role in cancer by either suppressing or promoting tumorigenesis in a context dependent manner. In this study we demonstrate for the first time that phosphorylation of profilin 1 at serine 137 enhances oncogenic properties in breast cancer cells. Inhibitors targeting profilin 1 phosphorylation directly or indirectly through inhibition of kinases that phosphorylate profilin could be valuable therapeutic agents that can alter its activity and thereby control the progression of cancer.

## Introduction

Breast cancer is the first most common cancer in women in USA (www.cdc.gov) and is predicted to overtake cervical cancer in women in India by 2020 [1]. Early detection and treatment is prolonging life expectancy in breast cancer patients. Mortality due to breast cancer has decreased significantly in the past decade; however, many patients die due to disease relapse and metastasis. While many nonmetastatic breast tumors are initially hormone dependent, most acquire hormonal autonomy at later stages to become metastatic [2]. Cell migration and invasion are important steps towards breast cancer progression and metastasis that require dynamic cytoskeletal and cell membrane rearrangement for cell motility. Profilin (PFN), an actin modulating protein, is an abundant cytoskeletal protein and widely distributed in eukaryotic cells [3]. Profilin and its isoforms are considered to be important regulators of the actin-based cytoskeleton. Under *in vitro* conditions, profilin in high amounts inhibits actin polymerization by sequestering actin monomer from F-actin or, inversely when present in low amounts promotes actin polymerization in appropriate circumstances [4]–[6]. It has been shown that profilin 1 promotes membrane protrusion, cell migration and invasion while profilin 2 suppresses these processes in breast cancer [7]. Its interaction with PI(4,5)P2 (Phosphatidylinositol 4,5-bisphosphate), a component of the phosphatidylinositol cycle, serves as a link through which the signaling pathways communicate with the dynamics of the actin cytoskeleton [8], [9]. Post-translational modifications (PTM), such as nitration and phosphorylation, of profilin 1 have biological implications [10]–[12]. Phosphorylation at serine residue (S137) allows it to interact with greater affinity to actin and proline rich proteins such as p85α, the regulatory subunit of PI3K (Phosphoinositide 3-Kinase), a lipid kinase with tumor promoting activity [12]. It is increasingly evident that profilin is associated with important signaling proteins known to be involved in transformation, such as PI3K (apoptosis) and Protein Kinase C (PKC) (tumorigenicity) [13], [14]. However, the role of serine phosphorylation of profilin in cancer remains to be explored.

Profilin 1 has been extensively studied in breast cancer cell lines and mouse models for breast cancer [14]–[16]. Immunohistochemical analysis of human breast cancers revealed intermediate to low levels of profilin 1 expression [16]. Differential expression of profilin was reported in different cancers suggesting the complexity in the role of profilin in cancer [17], [18]. Studies supporting its role in cellular proliferation, migration and invasion in breast cancer differed suggesting a dichotomous role for profilin 1 in breast cancer progression thereby necessitating further studies in this area. Among its different ligands, it is reported that profilin’s function as a tumor suppressor depends on its actin binding ability [17]. Our present data on human breast cancer tissues from Indian population revealed enhanced expression of profilin 1 when compared to adjacent normal tissues of the breast. Previous data from our lab demonstrated that phosphoprofilin (S137) binds to actin monomers with higher affinity and therefore might alter actin polymerization. Any change in actin polymerization affects cell migration, invasion and angiogenesis. A recent study demonstrated similar effects due to phosphorylation of profilin at tyrosine Y129 and its potential role in facilitating adult angiogenesis in tissue wound healing [10]. Phosphoprofilin (pY129) also facilitated glioblastoma progression by endothelial secretion of angiocrine factors that induced hypoxia inducible factor 1α (HIF1α) stabilization and accumulation in normoxic conditions [19]. We hypothesized that phosphorylation of profilin 1 at S137 could be crucial in breast cancer progression. Studies presented here demonstrate that S137 phosphorylation of profilin 1 not only augment migration and invasion in breast cancer cells (MCF7) but also enable them to become anchorage independent. Other mutations in profilin 1 that hinder profilin’s interactions with actin or poly-L-proline (PLP) containing proteins demonstrated enhanced tumorigenic properties indicating the significance of serine phosphorylation of profilin in cancer progression and the potential in making pS137 a chemotherapeutic target to curb breast cancer.

## Materials and Methods

### Subjects

Primary, non-invasive and invasive breast carcinomas following radical mastectomy at different tumor stages (T1-4N0-1M0) of female human subjects (27 ductal carcinoma and 3 lobular carcinoma cases) belonging to varying age groups from 40–70 years were used for the study. Normal tissue adjacent to the tumor from the same breast was used as control. The nature of the tissue was confirmed by histopathological studies. Prior written consent from patients was obtained before tissue samples were collected following mastectomy and the samples were not exclusively used for this study and not meant for commercial use. The study and the method of tissue collection were approved by the “Institutional Ethics Committee for Biomedical Research”, Hyderabad, India and Osmania University.

### Collection and processing of breast tissue samples

After surgical resection (mastectomy), samples were collected in ice-cold Tris- buffered saline (10 mM Tris, pH 7.2 and 0.9% NaCl) and immediately processed for future analysis. Tissues were ground in Remi homogenizer on ice in tissue lysis buffer (40 mM Tris buffer, pH 7.2, 1% Triton X-100, 10% glycerol, 1 mM β-ME, 2 mM EGTA, 1 mM EDTA, 130 mM NaCl, 50 mM NaF, 1 mM Na_3_VO_4_ and protease inhibitor cocktail) and centrifuged at 16000×g for 20 min at 4°C. The supernatant obtained was used for western blotting analyses.

### Isolation of primary epithelial cells from breast tissue

1 g of breast tissue (tumor and normal) derived after mastectomy was cut up manually into small pieces, approximately 0.5 cm cubed, collected and washed in ice-cold Kreb’s Ringer buffer (116 mM NaCl, 4.2 mM KCl, 2.5 mM CaCl_2_, 1.6 mM NaH_2_PO_4_, 1.2 mM MgSO_4_, 22 mM NaHCO_3_, 11 mM glucose, pH 7.4). The tissue was digested overnight at 37°C in a shaking incubator at 50 rpm with 0.5 mg/ml collagenase (Type IV, sigma) in 5 ml RPMI medium (RPMI 1640 media containing 2 mM L- glutamine, 100 U of penicillin/ml, 100 µg streptomycin/ml and 10% fetal calf serum). Following enzyme digestion, the fat layer was decanted by centrifuging at 1000×g for 5 min at room temperature (RT) and washed several times with the buffer. The cell-rich supernatant was backflushed through 60 µ and 50 µ meshes to isolate breast organoids from red blood cells, fibroblasts and endothelial cells. The cells were disaggregated by pipetting in media containing 0.25% trypsin-EDTA followed by filtration through 40 µ meshes. The cell pellet was collected and washed three more times to yield a predominantly single cell suspension [20]. The final pellet was resuspended in 1 ml media and the cells were counted on cytometer after trypan blue staining. The cells were lysed in M2 lysis buffer (20 mM tris, pH 7.6, 0.5% NP-40, 250 mM NaCl, 3 mM EGTA, 3 mM EDTA, 5 mM NaF, 5 mM Na_3_VO_4_ and 5 µg/ml protease inhibitor cocktail) and the lysate was cleared by centrifuging at 12000×g for 15 min at 4°C.

### Cell Culture

MCF7 cells were purchased from NCCS (National Center for Cell Science), Pune, India (previously published in [21]). MCF7 cells were grown in MEM supplemented with 0.01 mg/ml Insulin, 10% fetal bovine serum and 1% antibiotic-antimycotic solution. MCF7 cells transfected with profilin 1 and mutants were maintained in the above media supplemented with 800 µg/ml G418. Insulin and G418 were purchased from Sigma-Aldrich, USA.

### Western blot analyses

Total protein in clear supernatants obtained from breast tissue and cell lysates was estimated by Bradford’s method according to the manufacturer’s protocol (Sigma-Aldrich). 50 µg protein from tissue lysates and cell lysates was subjected to 10% tricine SDS-PAGE [22] and transferred onto nitrocellulose membrane. The membrane was blocked with 3% gelatin in TTBS buffer (10 mM Tris, 154 mM NaCl and 0.1% Tween-20, pH 7.5) for 1 hr, then washed with TTBS (4×10 ml) and incubated with rabbit anti-profilin 1 antibodies (1∶1000 dilution, Novus biologicals) or rabbit anti-PKCζ antibodies (1∶500, in-house) and anti-actin antibodies (1∶3000, Sigma) for 2 hr. After washings with TTBS, the blot was incubated with horse radish peroxidase conjugated secondary anti-rabbit/anti-mouse goat IgG for 2 hr. The blot was then colorimetrically developed using NBT/BCIP (Amersham Pharmacia Biotech).

### Sub-Cloning of GFP-Profilin 1 wild type (WT), S173A (phosphorylation mutant), R74E (actin mutant) and H133S (PLP mutant)

GFP-fusion constructs of profilin were constructed. GST clones of profilin-1 WT, R74E and H133S (characterized by [23], [24] were subcloned into EGFP-C1 vector using forward primer: 5′-AGC CAA GCT AGT GCC GGG TGG AAC GCC-3′ and reverse primer: 5′-CAG GGA TCC TCA GTA CTG GGA ACG CCG AAG-3′. Similarly, S137A from pcDNA3 construct [25] was subcloned into EGFP-C1 vector. All inserts were cloned between HindIII and BamH1 restriction sites (forward primer: 5′-CCA AGC TTC GAT GGC CGG GTG GAA CG-3′ and reverse primer: 5′-GCGGATCC CTA GTA CTG GGC ACG CCG A-3′). All the constructs including empty vector (eGFP-N1) were confirmed by plasmid sequencing (Invitrogen). These constructs were then overexpressed by transfection in MCF7 cells.

### Transfection and generation of cell lines

MCF7 cells were transfected with a control EGFP-N1 vector or following profilin1 expressing constructs: GFP-PFN1-WT (Wild-Type, human), GFP-PFN1-S137A (serine 137 phosphorylation mutant), GFP-PFN1-R74E (actin mutant) or GFP-PFN1-H133S (PLP binding mutant). For transfection, 0.5 µg plasmid DNA was mixed along with 1 µl of transfection reagent (chloroform:DT-DEAC lipid micelles made in laboratory) [26] in a total volume of 200 µl serum-free MEM media. The transfection mix was incubated at room temperature for 30 min, following which it was added to 20,000 MCF7 cells pre-incubated in serum-free MEM media for 1 hr. After 5 hr of transfection, media was changed to complete MEM media and cells were allowed to grow for 24 hr for protein expression. Selection media with 800 µg/ml G418 was added and polyclonal populations were selected over a period of two weeks.

### Cell Viability Assay

To determine cell growth of transfected clones, 2000 cells of each clone were plated in triplicate in 96-well plates and cell viability was assessed by MTT (3-[4,5-dimethylthiazol-2-yl]- 2,5-diphenyltetrazolium bromide) assay at 24 hr, 48 hr, 72 hr and 7 days. MTT reagent (diluted from a 4 mg/ml solution in PBS) was added to all the wells at a final concentration of 0.8 mg/ml and the cells were further incubated for 4 hr at 37°C. The reaction was terminated by adding 200 µl/well dimethyl sulfoxide (DMSO) and absorbance was read at 570 nm in an ELISA plate reader. Wells containing complete media alone was used as blank and the average values subtracted from the average values of all clones. The assay was repeated three times and analyzed using two-sided Student’s *t* test. Data points represent the mean±s.d and p<0.005 was considered significant.

### Soft agar growth assay

Anchorage independent growth was assayed by the soft agar growth assay as described elsewhere [27]. The first step involved plating a bottom layer of 0.6% agar in serum-free media in 12-well plates. The plates were incubated at room temperature for 30 min to solidify the agar. Cells were harvested by trypsinization and 1×10^4^ cells were mixed with 0.3% agarose (made in complete media) and layered carefully on the top of existing 0.6% agarose. The plates were covered with 1 ml of medium supplemented with 10% FBS and incubated at 37°C in a 5% CO_2_ incubator for 10days. Complete media was added every 2 days to the test wells. At the end of 10 days, cell colonies with >100 µm in diameter were imaged and counted under a microscopic field at 2.5× magnification. Mean colony count was based on numbers from 5 different fields imaged under 2.5× magnification from two independent experiments done in duplicate for each treatment condition and was analyzed using two-sided Student’s *t* test.

### Wound healing assay

5×10^4^ cells were plated in a 6-well plate in triplicate and allowed to grow to confluency. Cells were washed and left in serum-free media for 5 hr. A scratch was made and media was added that was either only serum-free or with 200 nM PDBu (phorbol 12,13-dibutyrate) or with 20 µM LY294002/200 nM PDBu. Images were captured at 10× magnification using Leica Microscope at zero hour and again at 24 hr. The scratch area at zero hour and empty area not covered by migrated cells after 24 hr was analyzed with ImagePro software. The assay was repeated three times. Data points represent the mean±s.d. from five fields of uncovered scratch area from three independent experiments and p<0.05 was considered significant.

### Cell Invasion assay

Invasion assay was performed as per the protocol published by Dasgupta et al [27]. MCF7 overexpressing profilin 1 and its mutant clones were harvested by trypsinization and 1×10^5^ cells were placed in the top chamber of boyden filters as untreated/treated with 200 nM PDBu or a combination of 20 µM LY294002 and 200 nM PDBu in serum-free media. The filters were pre-coated with 100 µg/ml ECM (BD Biosciences) and incubated for 4 hr at 37°C before adding cells and the assay was performed according to the protocol [27]. Invaded cells were stained with hematoxylin and images for 6 different fields per treatment were captured using 40× magnification using a Leica microscope. The assay was repeated twice in duplicate and analyzed using two-sided Student’s *t* test. Data points represent the mean±s.d. and p<0.05 was considered significant.

### Flow cytometry

Asynchronously growing MCF7 clones overexpressing profilin and its mutants were treated with hypotonic buffer (1 mg/ml sodium citrate and 0.3% Igepal) for 20 min. The buffer digests the cytoplasm and nuclei detach from the plate. Nuclei are gently pipetted a few times and collected in an eppendorf for Green Fluorescence acquisition [28]. Similarly, 10^5^ cells of each clone were plated and serum starved for 24 hr. Cells were then treated with DMSO control/200 nM PDBu/a combination of 20 µM LY294002 and 200 nM PDBu for 24 hr. Nuclei were harvested and subjected to flow cytometry for Green Fluorescence acquisition. The data was acquired with flowcytometer (Millipore Guava 8 HT easycyte). 50000 events were acquired with flow rate of 0.59 µl/s. Data was analyzed by FlowJo software. Experiments were done three times in duplicate.

### Immunofluorescence and Confocal Microscopy

MCF7 clones (40,000cells) were plated on cover slips (Corning) in a 6-well plate and cultured for 24 hr to visualize localization of overexpressed profilin 1 and its mutants. The cells were fixed in 2% para-formaldehyde for 15 min. The nuclei were stained with DAPI. Cells were mounted on glass slides. Images were captured at 63× oil immersion magnification with a Leica confocal microscope [29]. Images are representative of three independent experiments.

### SDS-PAGE Zymography

5×10^5^ cells were plated in a T25 flask and allowed to grow for a day. Cells were placed in serum-free media for 5 hr and then treated with either DMSO control media or 200 nM PDBu for 24 hr. Conditioned media were collected and centrifuged to remove debris. Samples were concentrated 10times using a centricon (Millipore, Bedford, MA) from 2 ml to 0.2 ml. Zymography was performed as described previously by Toth M et al., [30] with a little modification. Samples were loaded on a 10% SDS-polyacrylamide gel incorporated with 0.1% gelatin (Sigma) for electrophoresis. MMP2 and MMP9 zymographic standards were used as positive controls (Chemicon). After electrophoresis, MMPs were allowed to renature by washing the gel twice in 50 ml of 2.5% Triton X-100 for 30 min, incubated for 18 hr at 37°C in developing buffer, and stained for 1 hr with 0.5% Coomassie brilliant blue G250. After destaining gelatinolytic activity was visualized as a transparent band against a blue background. Gels were imaged using a digital imaging analysis system (Bio-Rad). The assays were repeated three independent times.

### Statistical Analysis

All data was analyzed statistically by two sided student’s *t* test in Microsoft Office 2007 and statistical significance was estimated. Densitometric analysis of western blots was carried out with ImageJ software. Flow cytometry data was analyzed using FlowJo software.

## Results

### Human breast cancer tissues and primary cells derived from them express high levels of profilin 1

**Figure 1:**
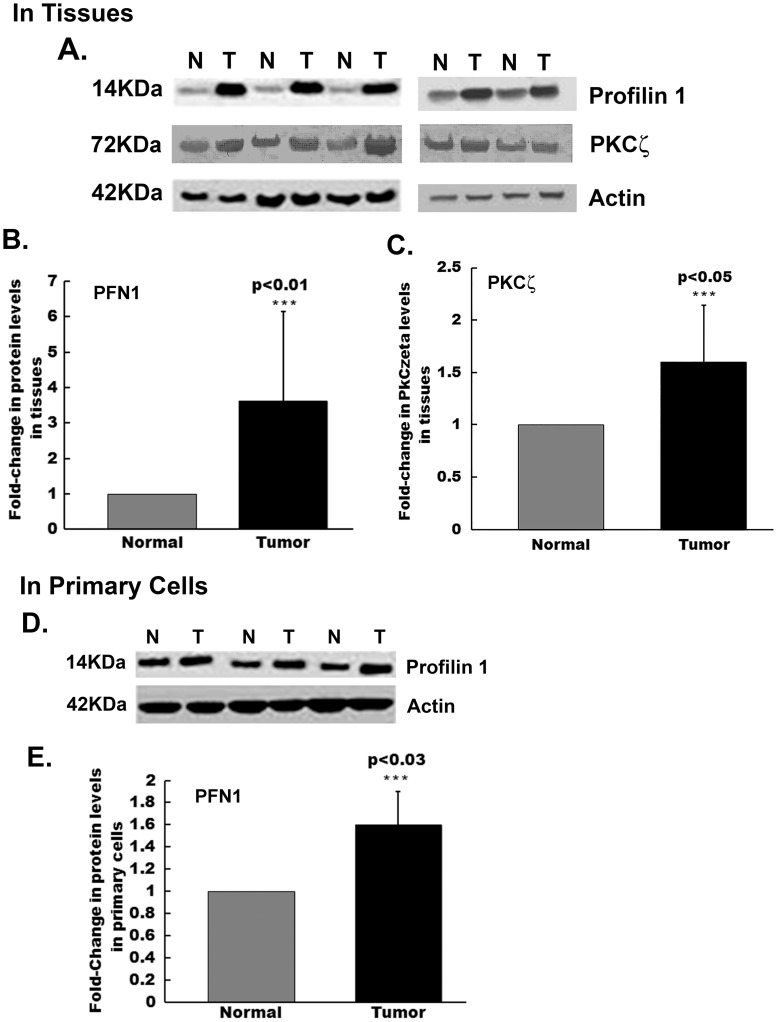
Profilin 1 and PKCζ levels are upregulated in human breast cancer tissue samples. (A) Representative western blot showing profilin 1, PKCζ and actin protein expression. Actin was used as loading control (n = 30). (B) Densitometric analysis revealed a 3.6±2.5-fold (***p<0.01) increase in profilin 1 levels and (C) a 1.6±0.5-fold (***p<0.05) increase in PKCζ levels in human breast cancer tissues compared to adjacent normal breast tissues. (D) Representative western blot showing profilin 1 levels in primary cells isolated from breast cancer and adjacent normal tissues. (E) Densitometric analysis reveals a 1.6±0.3-fold (***p<0.03) increase in primary cells from breast cancer tissues compared to normal adjacent breast tissues. N denotes adjacent Normal and T denotes Tumor tissues of the breast.

Published results on levels of profilin in different cancer tissues/cell lines are contradictory. Western blotting analyses was performed on lysates prepared from patient samples for breast cancer and adjacent normal breast tissues. Profilin 1 levels were significantly higher in breast cancer tissues compared to their adjacent normal counterparts (Figure 1A). Densitometric analysis of the western blots revealed profilin 1 was 3.6±2.5-fold (*p<0.01) elevated in cancer tissues (Figure 1B). Simultaneously, primary epithelial cells were isolated from the same patient samples and lysates were subjected to western blot analysis for profilin 1. A representative western blot is presented in Figure 1D and densitometric analysis of the western blots show a 1.6±0.3- fold (*p<0.03) increase in profilin 1 levels in cells isolated directly from the breast cancer tissues when compared to adjacent normal tissues (Figure 1E).

Post-translational modification such as phosphorylation can alter the function of cytoskeletal-associated proteins in tumorigenesis; since profilin 1 is known to undergo phosphorylation at Ser137 preferentially by PKCζ, an enzyme with potential role in breast cancer cell chemotaxis and tumor invasion [31]–[33]; we also examined PKCζ levels by western blotting in breast cancer and adjacent normal tissues (Figure 1A, middle panel). Figure 1C is a densitometric representation of increased PKCζ expression (1.6±0.5-fold, *p<0.05) in breast cancer tissues. Elevated protein levels of PKCζ in breast cancer tissues were indicative of a phosphorylation event targeting profilin 1 too. Therefore, we studied the probable role of profilin 1 phosphorylation at serine residue (S137) in breast cancer.

### Overexpression of profilin 1-wild type and profilin 1 mutants in breast cancer cell line, MCF7

**Figure 2:**
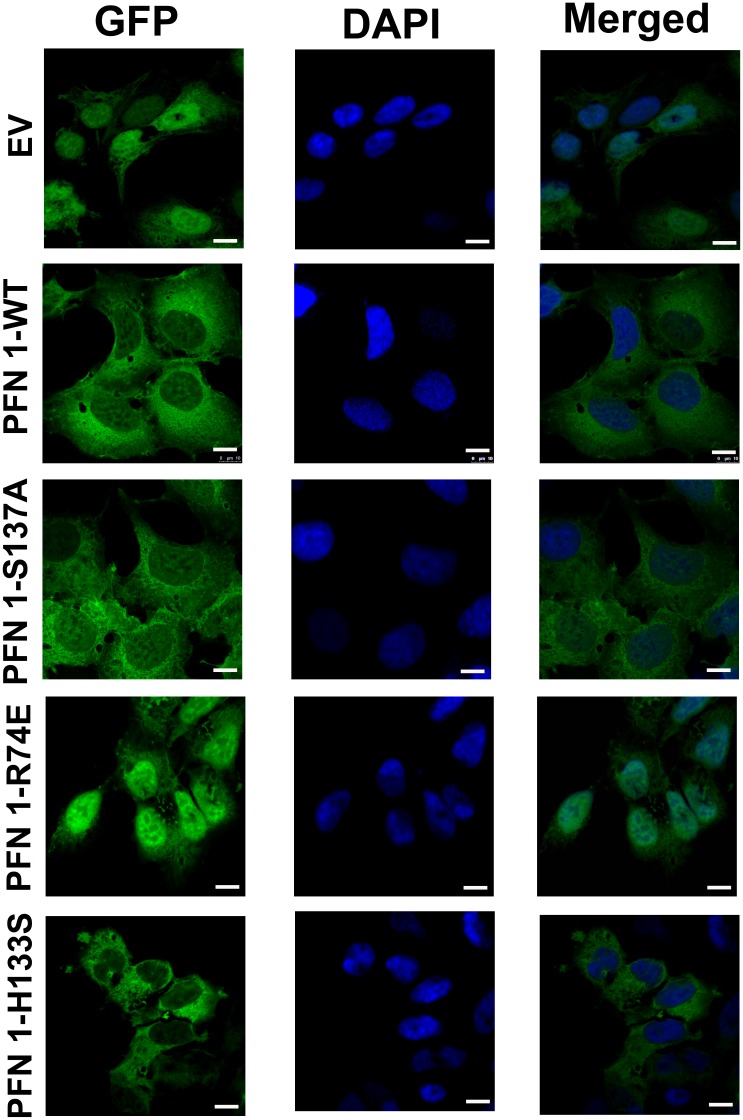
Overexpression and localization of profilin 1 and its mutants in MCF7 breast cancer cells. Left panel depicts images of GFP-Profilin and its mutants, middle panel shows DAPI images and right panel shows merged images for each clone. PFN-WT (profilin 1 Wild-type) and PFN-S137A (serine phosphorylation mutant) were distributed more or less evenly in the cytoplasm and nucleus. PFN-R74E (actin mutant) was predominantly nuclear and PFN-H133S (poly-L-proline mutant) was mostly cytoplasmic in expression. Magnification-63× and scale bar-10 µm.

**Figure 3:**
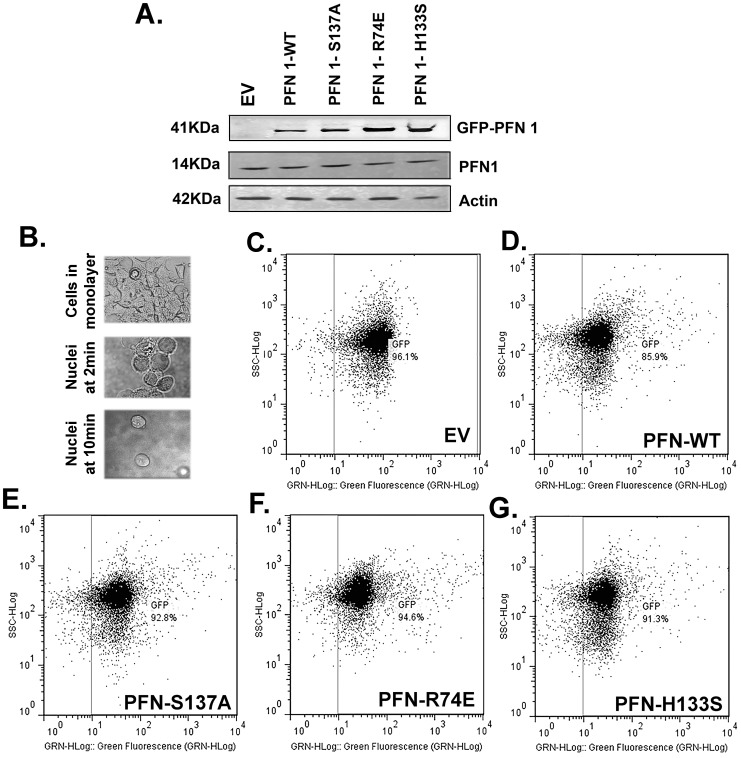
Flow cytometric analysis of profilin 1 clones and its mutants reveal overexpression of profilin. (A) Western blot showing overexpressed profilin 1 and its mutants (41 KDa), endogenous profilin 1 (14 kDa) and actin (42 KDa). (B) Phase contrast image showing well spread out monolayer of transfected MCF7 cells (upper panel) and rounded nuclei alone at 2 min and 10 min (middle and bottom panels). Magnification-20×. Flow cytometric analysis showing green fluorescent positive nuclei from (C) EV, (D) PFN-WT, (E) PFN-S137A, (F) PFN-R74E and (G) PFN-H133S clonal populations.

Since profilin 1 levels were high in patients with breast cancer, we overexpressed profilin 1 and some of its mutants in MCF7 cells to study their effect on cancer progression. MCF7 cells were transfected with each of the following vectors individually: EGFP-N1 (Empty Vector, designated as EV), GFP-Profilin 1-wild type (PFN-WT), GFP-Profilin 1-S137A (mutant for serine 137 phosphorylation site; PFN-S137A), GFP-Profilin 1-R74E (mutant for actin-binding site, PFN-R74E) and GFP-Profilin 1-H133S (mutant for poly-L-proline (PLP) sequence binding, PFN-H133S). Expression of profilin clones was studied by confocal microscopy. Left panel depicts images of GFP-Profilin and its mutants, middle panel shows DAPI images and right panel shows merged images for each clone (Figure 2). Each clone showed varying localization of GFP-Profilin. EV expression was observed in the nucleus and cytoplasm. PFN-WT, PFN-S137A and PFN-H133S showed cytoplasmic and nuclear expression. Of these, PFN-H133S was predominantly cytoplasmic and PFN-R74E was mostly nuclear with little cytoplasmic expression. These results indicate that interaction of profilin with actin is essential to maintain profilin in the cytoplasm. PFN-H133S showed the least expression of nuclear profilin suggesting that interaction with PLP-containing proteins could be essential for its nuclear translocation. Simultaneously, lysates were prepared from MCF7 cells expressing different clones of profilin and subjected to western blotting with anti-profilin antibodies. EV did not show over-expressed profilin whereas all the clones showed a band corresponding to GFP-profilin (41 kDa). Endogenous cellular profilin 1 (14 kDa) was observed in all the lanes including EV and actin was used as loading control (42 kDa) (Figure 3A). Next, we determined the nuclear expression alone of GFP-profilin and mutants in asynchronously growing MCF7 cells by subjecting them to hypotonic solution that bursts the cytoplasm but leaves the nuclei intact and detached from the plate. The nuclei are collected as a single cell suspension and subjected to flow cytometric analysis (Figure 3B). Untransfected MCF7 cells were used to gate the positive nuclei from the clones. All the clones showed similar numbers of nuclei that were positive for profilin 1. Figure 3C–3G show the flow cytometry dot plots of GFP-positive nuclei from asynchronously growing EV (96.1%), PFN-WT (85.9%), PFN-S137A (92.8%), PFN-R74E (94.6%) and PFN-H133S (91.3%) respectively. These clones were then selected in neomycin (G418) to obtain a 100% positive polyclonal population overexpressing profilin 1 and its mutants respectively.

### Overexpression of profilin 1 facilitates cell growth and anchorage independent growth that is further enhanced by its phosphorylation at Serine137

**Figure 4:**
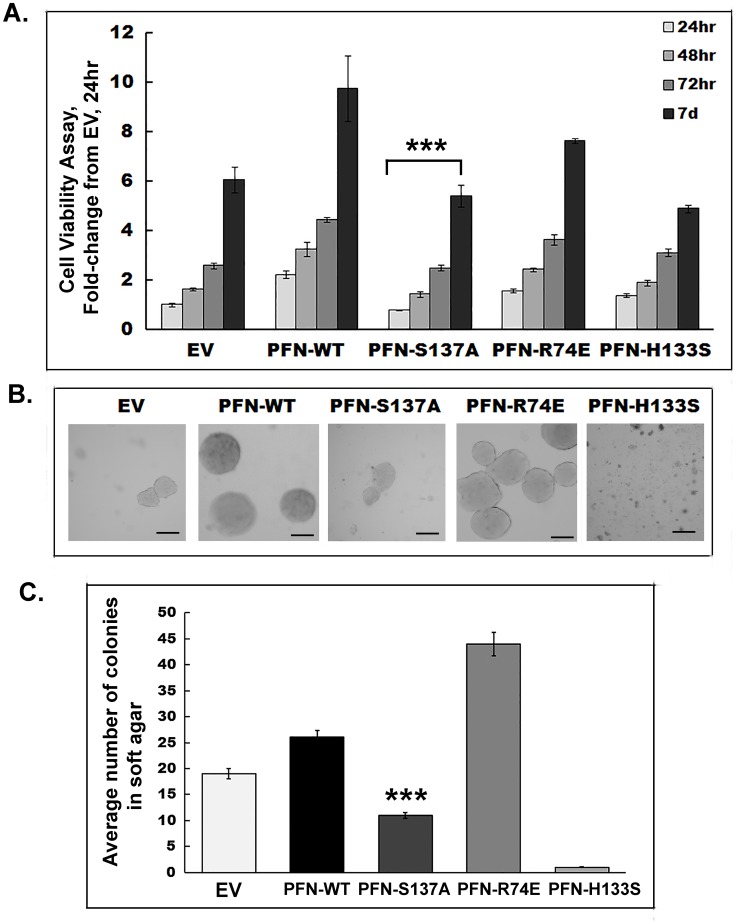
Overexpressed profilin 1 and its mutant clones exhibit different cell growth and show difference in soft agar colony forming ability. (A). Cell growth/viability (MTT assay) of MCF7 cells transfected with profilin 1 and its mutants demonstrate that PFN-WT had the highest viability followed by PFN-R74E, PFN-H133S and PFN-S137A at all time points studied-24 hr, 48 hr, 72 hr and 7days. PFN-S137A (***p<0.005) showed viability comparable to EV. (B). Representative phase contrast images from a soft agar colony formation assay demonstrating that PFN-R74E formed the most number of colonies followed by PFN-WT. PFN-S137A formed smaller and significantly less number of colonies comparable in size to EV. PFN-H133S did not form any measurable colonies. Magnification-20×. Sale bar-100 µm. (C) Histogram showing the average number of soft agar colonies from 5 different fields of MCF7 cells overexpressing profilin 1 and its mutants. PFN-S137A had the least number of colonies above 100 µm in size (***p<0.005). PFN-H133S did not grow in soft agar.

We first assessed the growth ability of transfected cells in culture at 24 hr, 48 hr, 72 hr and 7days of plating in complete media by MTT assay (Figure 4A). PFN-WT demonstrated the highest viability at all time points studied followed by PFN-R74E and PFN-H133S. PFN-S137A showed the least growth rate and was comparable to EV transfected MCF7 cells (***p<0.005). Anchorage independency is one of the hallmarks of cancer and this feature allows tumor cells to invade adjacent tissues and lead to metastasis [34]. To determine which of the profilin 1 mutants might interfere with the tumorigenic property of MCF7 cells, soft agar assay was performed and colonies above 100 µm where counted and plotted. Figure 4B shows representative images of colonies in soft agar of different mutants of profilin 1 and figure 4C is a graphic representation of the average number of colonies for each clone from 5 different fields under 2.5× magnification. We observed that MCF7 cells expressing PFN-WT formed significantly more and larger colonies in soft agar compared to EV-expressing MCF7 cells (**p<0.01). PFN-S137A formed smaller and less number of colonies than EV and 42 percent less number of colonies (***p<0.005) than PFN-WT, thereby indicating that (S137) phosphorylation of profilin 1 facilitates anchorage independency in breast cancer cells. Actin mutant (PFN-R74E) formed 70 percent more colonies (**p<0.01) than PFN-WT, consistent with previous reports demonstrating that profilin 1 defective in actin binding ability promotes tumorigenesis [17]. Defect in PLP binding ability of PFN (PFN-H133S) did not allow any colony formation in soft agar, though the viability of these cells in monolayer cultures was fairly comparable to PFN-R74E till 72 hr and decreasing by 7days. These results were in corroboration with Wittenmeyer’s study [17] reporting the inability of PFN-H133S to form tumors in mice.

### Phosphorylation of profilin 1 at S137 leads to enhanced migration and invasion

**Figure 5:**
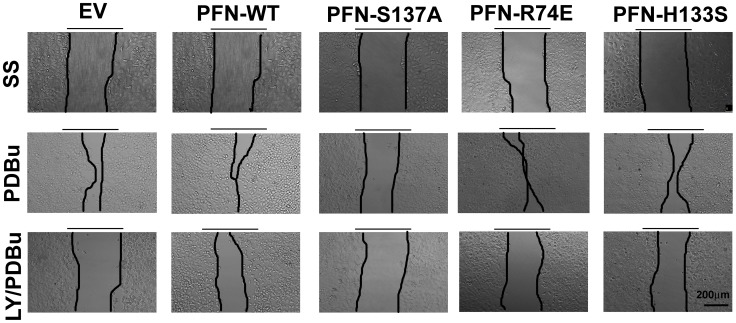
Phosphorylation of overexpressed profilin 1 increases the migratory ability of MCF7 cells. Upper panel images are representative of MCF7 cells overexpressing profilin 1 and its mutants in serum free media after 24(phorbol 12,13-dibutyrate) at 24 hr. Cells overexpressing PFN-WT, PFN-R74E migrated the most. PFN-H133S also migrated considerably. PFN-S137A showed least coverage of the wound. Lowest panel of images demonstrate that pre-treating cells with 20 µm LY294002 for 30 min prior to 200 nM PDBu for 24 hr prevented cells from migrating into the wound. Magnification-20×. Scale bar-200 µm.

**Figure 6:**
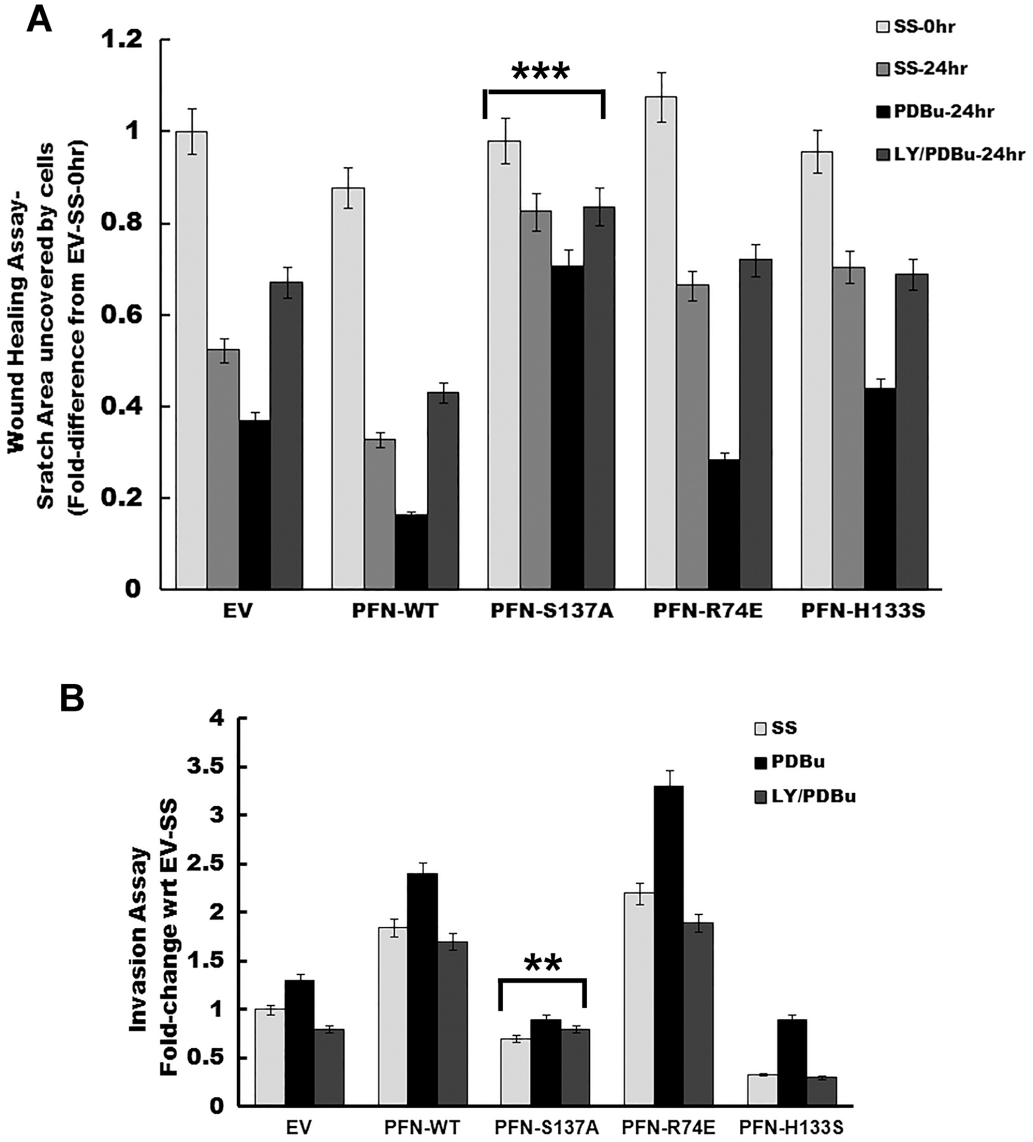
Phosphorylation of overexpressed profilin 1 increases migration and invasion in MCF7 cells. (A) Histogram showing the wounded area that was left uncovered by cells at 24 hr of making the scratch. Cells were either in serum-free media or supplemented with 200 nM PDBu or 20 µm LY294002/200 nM PDBu. Migration was observed in all conditions after 24 hr of scratch when compared to scratch at 0 hr of treatment. Cells treated with PDBu showed maximum migration. PFN-S137A did not show any significant difference in all conditions (***p<0.005). (B) Invasion assay by boyden chamber assay shows that PFN-S137A (**p<0.05) did not show any significant changes in invasion at 18 hr under any conditions tested, i.e., serum-free media or 200 nM PDBu or 20 µm LY294002/200 nM PDBu. PDBu treatment promoted invasion in all the clones. PFN-WT invaded more than EV, PFN-R74E showed maximum invasion and PFN-H133S the least. LY294002 inhibited invasion significantly in all the clones.

Whether phosphorylation of profilin 1 affects migration and invasion of breast cancer cells expressing various profilin 1 mutants was next assessed by wound healing assays and boyden chamber assays respectively. PKCζ activity can be reduced by the pharmacological inhibitors of PI3K. Previous *in vivo* studies from our lab have demonstrated that phorbol 12,13-dibutyrate (PDBu), a protein kinase C activator (PKC), phosphorylates profilin 1 at Serine 137 and the effect is reversed by LY294002, an inhibitor of PI3K and subsequently PKCζ [12], [35]. We studied their effect on profilin 1 mediated migration and invasion. MCF7 cells overexpressing profilin 1 and its mutants were grown to confluency and a scratch was made with a pipet tip. Images were captured at 20× magnification of the scratch at zero hour for each treatment. After 24 hr following treatments, images were captured and non-migrated area was analyzed. Figure 5 shows representative images of the wound and migrated cells into the wound for all the clones. All clones migrated to some extent into the wound at 24 hr in serum-free media. PDBu induced significant migration in all clones but not in the PFN-S137A mutant clone, therefore demonstrating that phosphorylation of profilin 1 at Serine 137 is an important event in promoting migration of MCF7 cells. PFN-WT and PFN-R74E expressing cells migrate more than EV expressing cells upon treatment with PDBu (**p<0.05). Pre-treatment of cells with LY294002 significantly abrogated migration of MCF7 cells overexpressing PFN-WT, PFN-R74E and PFN-H133S (**p<0.05). PFN-S137A did not show much change in migration at 24 hr in either serum-free media or PDBu or LY/PDBu treated cells (***p<0.005) (Figure 6A). This result reinforces the fact that serine phosphorylation of profilin 1 facilitates migration of breast cancer cells.

Similarly, invasion assays using ECM (extracellular matrix) coated boyden chamber filters revealed that PFN-WT and PFN-R74E cells invade more than EV cells in the presence and absence of PDBu. PFN-R74E showed more invasiveness than PFN-WT (**p<0.05). PFN-H133S invades the least (**p<0.05). However, PDBu- treated PFN-H133S showed 3 times more invasion when compared to serum-free media, indicating that though PFN-H133S is defective in binding to any PLP containing proteins, phosphorylation at Ser137 made it more aggressive. Pre-treatment with LY249002 inhibits invasion of all clones and in fact the invasiveness is reduced further than serum-free media. PFN-S137A demonstrates significantly reduced invasion even in the presence of PDBu (**p<0.05) (Figure 6B).

### Phosphorylation of profilin 1 induces MMP2 and MMP9 activity

**Figure 7:**
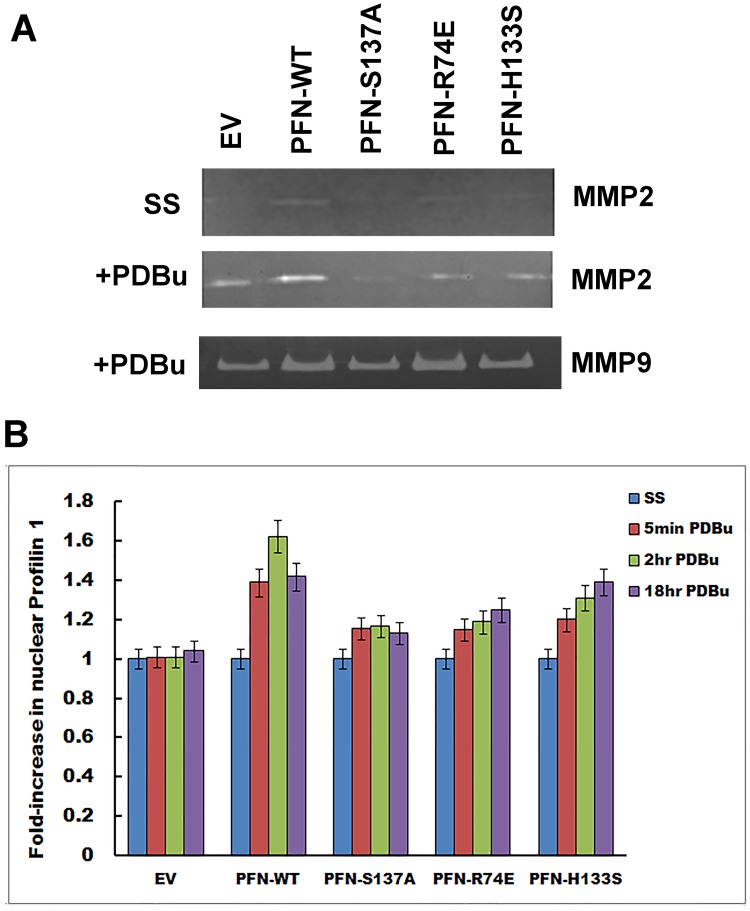
MMP2/MMP9 activity increases with phosphorylation of profilin 1 in MCF7 cells. (A) Gelatin zymography showing minimal MMP2 activity in serum-free media at 24 hr in all the clones. 200 nM PDBu treatment induced MMP2 and MMP9 activity significantly. PFN-WT expressed highest MMP2 activity whereas PFN-S137A the least. MMP9 activity was most expressed in PFN-R74E and PFN-WT followed by PFN-H133S. PFN-S137A showed the least MMP9 activity comparable to EV. (B) Nuclear translocation of profilin 1 and its mutants increases with time upon exposure to 200 nM PDBu when compared to serum starved cells except for PFN-S137A that remains constant throughout. PFN-S137A was comparable to EV.

Prior studies establish that Profilin 1 promotes MMP2 (Matrix metalloproteinase) secretion, ECM degradation and 3D morphogenesis in vascular endothelial cells [36]. Studies have also been reported showing colocalization of profilin with MT1-MMP (MMP14) at the leading edges of activated monocytes migrating on a layer of activated endothelial cells [37]. MT1-MMP14, in turn, activates MMP2 and MMP9. In the present study, we make evident that phosphorylation of profilin 1 at S137 plays a crucial role in promoting migration and invasion by activating MMP2 and MMP9. Conditioned media from clones growing in serum-free media or 200 nM PDBu containing media were subjected to zymography to analyze MMP2 and MMP9 activity. We found that serum-free clones produce very low MMP2 only, whereas PDBu-treated cells produced significant amounts of MMP9 and to a certain extent MMP2 also. Among the clones, PFN-WT showed maximum MMP2/9 activity. PFN-R74E also expressed increased MMP9 activity comparable to PFN-WT. PFN-H133S by itself was not very migratory and invasive in nature but PDBu-treatment makes this clone sufficiently pro-invasive. Consistent with that data, we found MMP2/9 activity increasing in this clone in response to PDBu. PFN-S137A had the least MMP2 activity and remained same under all conditions tested. MMP9 activity in PFN-S137A was comparable to EV and considerably less than PFN-WT (Figure 7A).

### Phosphorylation increases translocation of a subset of profilin 1 to the nucleus

Though profilin 1 has been shown to be localized partially in the nucleus, very few studies are available on its role in the nucleus. Recent studies carried out in dipteran *Chironomas tentans* have shown that a fraction of profilin in located in the nucleus; is highly concentrated in the nucleoplasm and nuclear periphery. It associates with protein-coding transcriptionally active genes and is independent of actin [38]. Similarly, we observed PFN-WT and its mutants to be localized in the nucleus, especially PFN-R74E, mutant for actin-binding was predominantly nuclear. We also find that increasing amounts of profilin localizes to the nucleus upon phosphorylation by PDBu as early as 5 min and upto 18 hr when compared to serum starved cells (Figure 7B). PFN-WT showed the most nuclear concentrations of profilin 1 when compared to serum starved cells. However, PFN-S137A, mutant for phosphorylation at serine 137, did not show any change in profilin localization with or without PDBu (Figure 7B). These results suggest that phosphorylation of profilin is importing profilin into the nucleus and probably facilitating in the binding of profilin to transcriptionally active genes relative to promoting tumorigenesis. This observation will need more confirmatory studies and identification of nuclear proteins or genes regulated by profilin.

## Discussion

In the present study, we report elevated profilin 1 expression in non-metastatic, primary breast carcinoma tissues from Indian population. Profilin levels have been found to be elevated in some pathological states such as adipose tissue inflammation, liver and kidney diseases [39]–[41] though in cancers the reports have been ambiguous. Profilin 1 was found to be downregulated in breast cancer cells CAL51 [16], in pancreatic cancer [42], and in hepatocarcinoma tissues [43] and upregulated in gastric cancer [44]. Analysis of N1E-115 neuroblastoma cell line for differentiation related cytoskeletal proteins also indicated profilin 2 to be elevated in expression [45]. Profilin 1 was identified as a secreted protein from tumor masses at progressive stage in skin fibromas [46], in oral cancer saliva proteome [47] and also in pancreatic cancer secretome [42]. Whether elevated profilin 1 levels alone are indicative of increased motility and aggressive cancers is debatable. Various cell lines from different cancers behaved differently with overexpression or abrogation of profilin 1 with respect to cell migration, invasion and proliferation [16], [18], [48], [49]. Recent studies report that profilin 1 is a potential biomarker for bladder cancer aggressiveness and suppressing its expression in T24 bladder cancer cell line decreased their motility with a concomitant decrease in actin polymerization [18]. Breast cancer MDA-MB-231 cells showed reduced migration upon overexpression of profilin 1 [48], however, an in depth analysis carried out by silencing profilin 1 showed suppressed endothelial cell proliferation, migration and cord morphogenesis [50]. Likewise studies involving upregulation of profilin showed increased invasiveness and growth of smooth muscle cells [51] and also resulted in glomerular cell proliferation [41]. These observations support the notion that profilin is directly associated with cell motility, migration and proliferation. Hence the need to investigate the different aspects that regulate profilin’s function in breast tumorigenesis and cancer progression.

Earlier studies indicate profilin 1’s involvement in cell motility in the intracellular movement of pathogenic organisms [52] and migration of lower eukaryotic organisms such as *Dictyostelium* and *Listeria* [53], [54]. It is known that the profilin-actin complex is recruited at the barbed end of actin filaments at the protruding end of lamellipodia in migrating cells where actin polymerization is initiated. Impaired motility of intracellular pathogens was observed in profilin-deletion experiments and revealed the importance of profilin in bacterial motility [52], in migration and proliferation of lower eukaryotes [53]. Profilin-deficient mice were embryonically lethal and profilin 1 null mice embryos did not survive indicating its necessity in cell division and survival during embryogenesis [55]. Profilins assist F-actin elongation at the leading edge of migrating cells through their interactions with a host of actin-binding proteins like Ena (enabled)/VASP (vasodilator stimulated phosphoprotein) [56], [57]. Profilin 1 and profilin 2 protein ratios in a cell can also determine the extent of actin polymerization and cellular motility [7]. Previously known facts include that both actin and polyproline interactions of profilin 1 are essential for tumorigenesis and enhanced migration/invasion [17], [36]. Studies published recently report that phosphorylation of profilin at tyrosine 129 in endothelial cells facilitates secretion of angiocrine factors. These, in turn, stabilize HIF-1α in a hypoxia-independent manner to promote glioblastoma progression [19]. Through our studies, we add a new dimension to the role of profilin in cell motility, invasion and aggressiveness. Phosphorylation at Ser137 of profilin 1 confers enhanced motility to MCF7 cells by itself irrespective of its actin binding or (PLP) protein binding capabilities.

**Figure 8:**
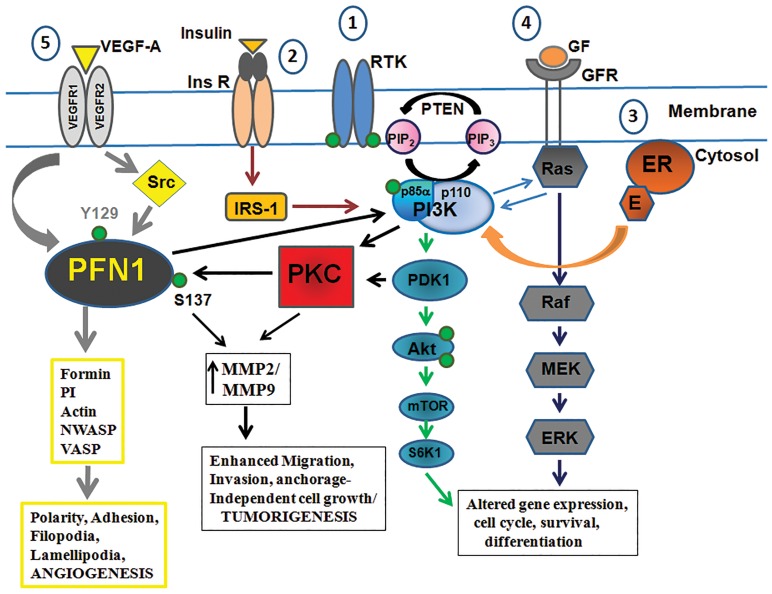
Schematic showing various pathways that triggers PI3K activity in breast cancer. These pathways can potentially lead to phosphorylation of profilin 1: (1) Receptor tyrosine kinases (RTK), (2) Insulin (Ins) and Insulin-like growth factors, (3) Estrogen receptor/Estrogen complex (ER/E), (4) Growth factors (GF) and (5) Vascular endothelial growth factors (VEGF-A) are well known pathways that activate PI3K (Phosphoinositide-3-Kinase) and most common to hormone-dependent and hormone-independent breast cancers. PI3K can in turn activate PKC’s (Protein Kinase C and its isoforms) including PKCζ. Among all PKC isoforms, PKCζ preferentially phosphorylates profilin 1 on Serine (S137) residue that leads to enhanced tumorigenic properties in breast cancer cells. VEGF-A can also lead to tyrosine phosphorylation of profilin 1 and increases angiogenic activity. It can be hypothesized that together these post-translational modifications could be instrumental in breast cancer progression.

Dynamics of profilin’s interactions with its ligands, including actin polymerization changes upon PTM such as nitration and phosphorylation [12],[58]. A number of signaling pathways triggered by external factors such as hormones (estrogen) (reviewed in [59]), growth factors (Insulin, Insulin-like growth factors) [60], radiation [61] activate PI3K which is known to activate PKC’s including the atypical PKCζ and therefore could possibly lead to phosphorylation of profilin 1 at S137 (Figure 8). Whether all these pathways eventually culminate to phosphorylate profilin 1 needs further investigation but a probable regulatory loop might be existing wherein profilin 1 activates PI3K [62] through its association with p85α subunit [63] which in turn increases the production of D3 phosphoinositides like PI(3,4,5)P3 (PIP3) known to activate PKC’s and Akt pathway. PKC’s trigger survival, motility and breast cancer progression (reviewed in [64]). We found PKC elevated in the breast cancer samples and another study found increased PI3K levels in breast cancers [65] indicating that profilin 1 phosphorylation could be an obligatory signaling event in promoting breast tumorigenesis. ROCK (Rho-associated Kinase) and PP1 (Protein phosphatase 1) are also known to regulate phosphorylation and dephosphorylation of profilin 1 at Ser137 [25]. Multiple kinases affect profilin phosphorylation thereby increasing the complexity in the nature of profilin 1’s function; its interactions with its many isoforms and oligomers [35]. These in turn will affect the cascade of protein interactions further and the biological consequences thereafter. From our studies in breast cancer cells using serine phosphorylation mutant of profilin (PFN-S137A), it is evident that profilin phosphorylation is crucial in promoting tumorigenesis. PFN-S137A overexpressing cells also showed significantly reduced growth, migration, invasion and colony formation compared to PFN-WT.

MMP9-mediated gelatin matrix degradation showed substantially higher expression in PDBu-stimulated breast cancer cells expressing PFN and its mutants studied. MMP2-mediated gelatin degradation was relatively lower than MMP9 in PDBu-stimulated cells. MT1-MMP (MMP14) has been shown to colocalize with profilin in monocytes at leading edges [37]. It is well known that MT1-MMP self-activates and in turn activates MMP2 and MMP9 also. Together these are involved in degrading extracellular matrix and making cells more motile and invasive. PFN-S137A expressing cells demonstrated least activity of MMP9 and MMP2 thereby suggesting that profilin phosphorylation is in part associated with activating MMPs, consecutively facilitating cellular mobility. PDBu can stimulate MMP14 and inhibiting PKCζ could reduce recruitment of MMP9 to podosomes and its release and activation [66]. The coordinated action of PI3K, PKC and profilin phosphorylation to induce MMPs needs further investigation to characterize the sequence of events in breast cancer progression. Another interesting finding was phosphoprofilin’s translocation to the nucleus that requires in-depth analysis focusing on the function of profilin in the nucleus and subsequent role in breast cancer progression. Very little information is available on nuclear profilin’s role in mammalian cells. Both cytoplasmic and nuclear distribution of profilin has been observed in cultured mammalian cells [67], [68]. Recent evidence from Skare et al. [69] suggests that profilin-actin complexes may play a role in pre-mRNA processing within the nucleus, due to extensive co-localization of profilin with the small nuclear ribonucleoprotein (snRNP)-associated Sm proteins and Cajal bodies. It is believed that mammalian embryogenesis requires nuclear and cytoplasmic profilin to maintain normal embryo development by interacting with actin-related proteins such as Arp 3, VASP, Dia1 and p80 coilin [70]. It would be interesting to demonstrate the importance of nuclear profilin and nuclear phosphoprofilin in breast cancer progression and targeting profilin 1 phosphorylation for cancer therapy could be a prudent option.
