# Correction: S137 Phosphorylation of Profilin 1 Is an Important Signaling Event in Breast Cancer Progression

**DOI:** 10.1371/journal.pone.0114164

**Published:** 2014-11-18

**Authors:** The PLOS ONE Staff

In the Funding section, there is an error in the fellowship for author Deepshikha Sharma. Dr. Sharma is is supported by a senior research fellowship from UGC (University Grants Commission) rather than CSIR. The correct Funding statement is: “This work was financially supported by UGC-MRP (India) funding to SSS. WR received a fellowship from UGC (University Grants Commission). DS is supported by a senior research fellowship from UGC, DJR from UGC and NBO from ICCR (Indian Council for Cultural Relations Fellowship). The funders had no role in study design, data collection and analysis, decision to publish, or preparation of the manuscript.”

In the Materials and Methods section, there is an error in the title of the sixth paragraph. The correct title is: “Sub-Cloning of GFP-Profilin 1 wild type (WT), S137A (phosphorylation mutant), R74E (actin mutant) and H133S (PLP mutant).”

In Figure 5, the panel for SS/PFN-WT is incorrectly shown as a duplicate of panel SS/EV. The correct version of Figure 5 can be viewed below.

**Figure 5:**
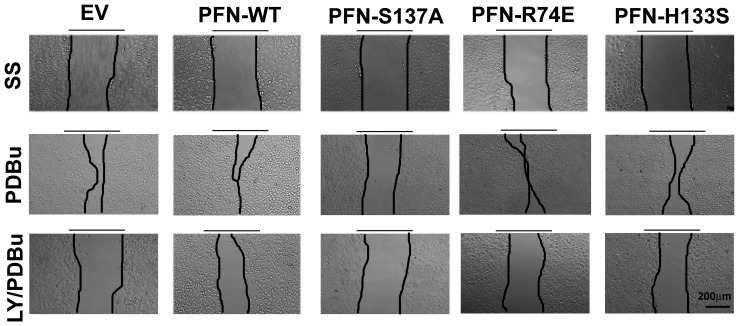
Phosphorylation of overexpressed profilin 1 increases the migratory ability of MCF7 cells. Upper panel images are representative of MCF7 cells overexpressing profilin 1 and its mutants in serum free media after 24(phorbol 12,13-dibutyrate) at 24 hr. Cells overexpressing PFN-WT, PFN-R74E migrated the most. PFN-H133S also migrated considerably. PFN-S137A showed least coverage of the wound. Lowest panel of images demonstrate that pre-treating cells with 20 µm LY294002 for 30 min prior to 200 nM PDBu for 24 hr prevented cells from migrating into the wound. Magnification-20×. Scale bar-200 µm.
